# Enhancement of Functional and Flavor Attributes of Soy Protein Isolate–Chitosan Coacervates via Ultrasonic Processing

**DOI:** 10.3390/foods15010025

**Published:** 2025-12-22

**Authors:** Jing Li, Can Luo, Changchun Li, Bin Li

**Affiliations:** 1 College of Life Science and Technology, Hubei Engineering University, Xiaogan 432000, China; 2 College of Food Science and Technology, Huazhong Agriculture University, Wuhan 430070, China; 3 Hubei Key Laboratory of Resource Utilization and Quality Control of Characteristic Crops, Xiaogan 432000, China

**Keywords:** coacervate complex, ultrasound, beany flavor, functional properties

## Abstract

Soy protein isolate (SPI)-based foods were limited by beany flavors and suboptimal emulsifying performance. Conventional flavor-mitigation strategies were often accompanied by losses in protein functionality, and the potential of ultrasound to simultaneously modulate structure, functionality, and flavor release of SPI–chitosan (SPI–CS) coacervates remained unclear. In this study, SPI–CS complexes were prepared and subjected to ultrasound at amplitudes of 20%, 50%, and 80% for 2 min, 5 min, or 10 min, after which their physicochemical properties, emulsifying properties, and the release of beany-flavor compounds were characterized. Coacervation with CS was found to increase particle size, decrease solubility, and enhance emulsifying indices while reducing beany-flavor release relative to SPI alone. Ultrasound treatment further decreased particle size, increased the absolute ζ-potential and surface hydrophobicity, and induced changes in secondary structure that were associated with improved solubility and emulsifying properties. Moderate amplitudes (20%, 50%) were more effective in enhancing emulsifying activity and alleviating flavor release, whereas prolonged treatment at 80% amplitude resulted in partial reaggregation and compound-dependent flavor behavior. Overall, ultrasonic processing was demonstrated to be a tunable, green strategy for engineering SPI–CS coacervates with concurrently improved functional and flavor attributes.

## 1. Introduction

Plant-based foods have been widely recognized in the food sector due to increasing interest in healthy lifestyles and environmental sustainability. However, the flavor profile of plant-based foods was impacted by the beany flavor and resulted in the consumption limitation of the plant-based foods industry. Although conventional approaches-including thermal processing, enzymatic treatment, and chemical modification-alleviated beany flavors to some extent, they remained limited by inefficiency, process complexity, and impaired protein functionality [1]. Thus, it remains challenging to develop a green, safe, simple, and efficient strategy capable of simultaneously improving the volatile profile and functional properties of plant-based proteins. Soy protein isolate (SPI) was widely utilized because of its balanced amino acid composition, abundant availability, and desirable functional attributes. It was previously demonstrated that chitosan (CS) substantially decreased the release of beany flavor compounds from SPI via electrostatic interactions, resulting in distinctly altered flavor profiles before and after treatment. In addition, CS could markedly enhance the functional properties (emulsifying property and antioxidant capacity) of SPI via the electrostatic interaction [2]. However, the solubility of SPI was substantially reduced when interacting with CS, which consequently limited its application. Therefore, an appropriate modification technology was required to further improve the functional attributes of the SPI–CS complex and enhance its ability to mitigate undesirable beany flavors.

Ultrasound, as an innovative, green, and efficient processing technology, has been widely utilized to promote macromolecular interactions and is favorable for the functional improvement of binary complexes [3,4]. Compared with traditional thermal or enzymatic modifications, which can reduce beany-flavor release but may impair protein functionality or increase processing complexity, ultrasound provides an environmentally friendly and tunable alternative [5]. On the one hand, ultrasound could enhance the binding affinity between the protein and flavor compounds, which could regulate the release of flavor compounds. It was reported that the off-flavor compounds in mung bean-based milk, including pentanol, hexanol, and hexanal, were significantly reduced after ultrasonic treatment [5]. In addition, synergistic ultrasonic-thermal treatments significantly decreased the release of beany flavor compounds of SPI [6]. On the other hand, structural properties of protein–polysaccharide coacervate complexes could be modified to improve their functional characteristics by ultrasound recently. The interactions (hydrogen bonding, electrostatic adsorption, and hydrophobic interactions) between molecules could be increased by the cavitation and high shear forces induced by ultrasound [7]. Ultrasonication further induced protein unfolding and exposed internal hydrophobic residues by altering protein–polysaccharide interactions. It was documented that the emulsifying properties of the complex between soluble soybean polysaccharide and soybean peptide aggregates were enhanced after ultrasound treatment [8]; similar improvements are observed for pea protein and high-methoxyl pectin complexes [9]. Moreover, ultrasound treatment could obviously improve the encapsulation efficiency of resveratrol by zein–chitosan coacervates [10]. Nevertheless, there is currently little information available regarding the impact of ultrasonication on the release behavior of beany flavor compounds from SPI–CS coacervate complex.

In this study, the effects of ultrasonic amplitude and duration on the emulsifying characteristics and beany-flavor release of the SPI–CS were systematically investigated. The physicochemical and structural properties of ultrasonicated SPI–CS coacervates were analyzed, including the particle size, ζ-potential, surface hydrophobicity, fluorescence spectroscopy, and the Fourier transform infrared spectroscopy. In addition, the beany-flavor release concentration was assessed both qualitatively and quantitatively. The findings could provide a valuable guide to the reduction in beany flavor of plant-based proteins and the theoretical basis for accurately regulating the flavor of plant-based foods.

## 2. Materials and Methods

### 2.1. Materials

Soy protein isolate (SPI, SUPRO-787) was purchased from Solae company (St. Louis, MO, USA) and its composition was reported previously [11]. 2-Methyl-3-heptanone (>95%) and the 8-anilino-1-naphthalenesulfonic acid were from Aladdin Reagent Co., Ltd. (Shanghai, China). Chitosan (CS, 100 kDa) was supplied by Macklin Biochemical Co., Ltd. (Shanghai, China). All other chemicals, which were of analytical grade, were procured from Sinopharm Chemical Reagent Co., Ltd. (Shanghai, China).

### 2.2. Preparation of SPI–CS Complex Treated by Ultrasound

The SPI solution (0.1 g/mL) was prepared in distilled water and stored at 4 °C overnight. The chitosan (100 kDa, 1% *w*/*v*) was dissolved in acetic acid (1%, *w*/*v*), and then a homogeneous solution was prepared. Then, the above solutions were mixed at a ratio of 30:1 (*v*/*v*) (SPI solution/chitosan solution) to obtain an SPI–CS dispersion with a total biopolymer concentration of 0.05 g/mL. Subsequently, the mixture was stirred until homogeneous, and the pH was adjusted to 7.0 ± 0.05 using 2 M NaOH. Then, the resulting SPI–CS mixture was treated using an ultrasonic device (FB705, Thermo Fisher Scientific, Waltham, MA, USA) equipped with a 12 mm titanium probe, with the sample immersed in an ice–water bath to maintain the temperature below 10 °C during ultrasonication. SPI–CS dispersions were ultrasonicated at amplitudes of 20, 50, or 80% for 2, 5, or 10 min (5 s on/off), generating nine ultrasonicated samples (three amplitudes × three times), in addition to the SPI control and the non-ultrasonicated SPI–CS sample. For each condition, three independent batches were prepared and processed separately. The dispersions were lyophilized to constant mass in a laboratory freeze-dryer (BT48, Millrock, Windham, NY, USA) under high vacuum for approximately 48 h to obtain powdered samples for subsequent analyses. Corresponding samples were designated as A-20%, A-50%, and A-80%, respectively, based on the ultrasound amplitude used. Additionally, the freeze-dried SPI–CS complex without ultrasonication was labeled as SPI–CS, and the raw SPI was marked as Control. Unless otherwise specified, analytical dispersions were prepared by redispersing the freeze-dried SPI, SPI–CS, or ultrasound-treated SPI–CS powders in the corresponding buffer or solvent to the desired concentration.

### 2.3. Particle Size and ζ-Potential

The ζ-potential and particle size of the samples (1 mg/mL with 10 mM PBS (pH 7)) were determined using a Nano-ZS laser analyzer (Malvern Instruments Ltd., Malvern, UK), and all measurements were conducted in triplicate at 25 °C.

### 2.4. Surface Hydrophobicity (H_0_)

The H_0_ of the samples was measured according to Li et al. [2]. Briefly, 50 μL of 8-anilino-1-naphthalenesulfonic acid (ANS) solution (2.4 mM in PBS (10 mM, pH 7)) was added to 5 mL of each diluted sample (0.01–0.2 mg/mL) prepared in the same PBS. The mixture was then incubated for 5 min under dark conditions. Fluorescence measurements were conducted with excitation and emission wavelengths at 390 nm and 470 nm, respectively.

### 2.5. Fluorescence Spectroscopy

The fluorescence spectra of the samples (1.0 mg/mL in PBS (10 mM, pH 7)) were recorded using a fluorescence spectrophotometer (F-4600, Hitachi, Tokyo, Japan). The instrument settings were as follows: the excitation wavelength of 290 nm, emission scan from 300 to 450 nm, and both excitation and emission slits of 5 nm. The H_0_ was obtained from the initial slope of fluorescence intensity versus sample concentration, where an increased slope reflects greater exposure of hydrophobic patches accessible to the ANS probe at the particle surface.

### 2.6. Fourier Transform Infrared Spectroscopy (FTIR)

A FTIR spectrometer, Nicolet NEXUS-470 (Madison, WI, USA), was employed to acquire the infrared spectra of the samples investigated. The lyophilized samples were mixed with KBr at a 1:100 (*w*/*w*) ratio and subsequently compressed into pellets for spectral analysis. The scans were conducted from 400 to 4000 cm^−1^, with each sample subjected to 32 repetitions at room temperature (25 °C), and a resolution of 4 cm^−1^. Secondary structure analysis was conducted using PeakFit 4.12 software to process the spectral data.

### 2.7. Free Sulfhydryl (F-SH) Content

The free sulfhydryl content was determined based on a protocol reported previously [11] without alteration. Samples were prepared by dispersing the aforementioned freeze-dried powders in Tris-Gly buffer (86 mM Tris, 90 mM glycine, and 4 mM EDTA) to a consistent protein concentration. Subsequently, 5 mL of each sample was combined with 50 μL of Ellman’s reagent (5,5′-dithiobis-(2-nitrobenzoic acid), DTNB) and incubated in the dark for 1 h. For comparison, a blank solution lacking DTNB was prepared. Then, the absorbance of the solutions prepared as mentioned above was measured at 412 nm. The sulfhydryl content of SPI was calculated according to the following relationship:

SH (μmol/g) = 73.53 × A_412_ × D/C

where A_412_ represents the absorbance at 412 nm, C is the sample concentration (mg/mL), and D is the dilution factor.

### 2.8. Solubility Measurements

The solubility of the samples was assessed based on the report [12]. The samples (1.0 mg/mL in PBS (10 mM, pH 7)) were centrifuged at 10,000× *g* for 20 min, after which the protein content in the supernatant was determined using the bicinchoninic acid (BCA) assay. Protein solubility was calculated as the percentage of supernatant protein relative to the total protein in the initial solution.

### 2.9. Volatile Compounds (Flavors) Analysis

Volatile compounds were identified using HS-SPME-GC-MS (Headspace Solid-Phase Microextraction Coupled with Gas Chromatography-Mass Spectrometry) on an Agilent 8890 GC system coupled with a 7000D MS system, employing an HP-5MS capillary column (Santa Clara, CA, USA). The analytical methodology followed our established protocol [11]. In brief, 10 mL of each sample (0.15 g/mL in PBS (10 mM, pH 7)) was incubated at 50 °C for 30 min and subsequently subjected to a 30 min SPME adsorption period. The GC program started with a 3 min hold at 50 °C, followed by heating to 160 °C at 3 °C/min with a 3 min hold, and then a final increase to 230 °C at 10 °C/min, maintained for 10 min. Identification of these flavor compounds was achieved using the NIST mass spectral library, and quantification was performed relative to the 2-methyl-3-heptanone internal standard.

### 2.10. Emulsifying Properties

The emulsifying properties of the samples, including the emulsifying activity index (EAI) and emulsifying stability index (ESI), were assessed according to previously reported methods [11]. The emulsions were created by mixing 15 mL (0.2%, *w*/*w* in PBS (10 mM, pH 7)) of sample solution with 5 mL of medium-chain triglyceride (MCT) and homogenized by ultra-turrax T25 homogenizer (IKAL, Staufen, Germany) at a speed of 10,000 rpm for 1 min. Then, 50 μL of emulsion was combined with 5 mL of SDS (0.1%, *w*/*w*). Subsequently, absorbance readings were taken at 500 nm. The two quantities were calculated as follows:

EAI (m^2^/g) = (2 × 2.303 × A_0_ × 100)/(C × L×0.25 × 10^4^)

ESI (%) = A_30_/A_0_ × 100%

where C is the protein concentration (g/mL), A_0_ and A_30_ denote the absorbance at 0 and 30 min, respectively, and L is the optical path length (1 cm).

### 2.11. Statistical Analysis

All experiments were performed in triplicate, and the data were reported as mean ± standard error. Significant differences among samples (*p* < 0.05) were determined using one-way ANOVA (SPSS v25.0) followed by multiple comparisons of means. Treatments sharing different letters in figures and tables are significantly different at *p* < 0.05. All figures were generated with Origin 2024.

## 3. Results and Discussion

### 3.1. Particle Size and ζ-Potential of SPI-CS Under Ultrasonic Treatment

**Figure 1:**
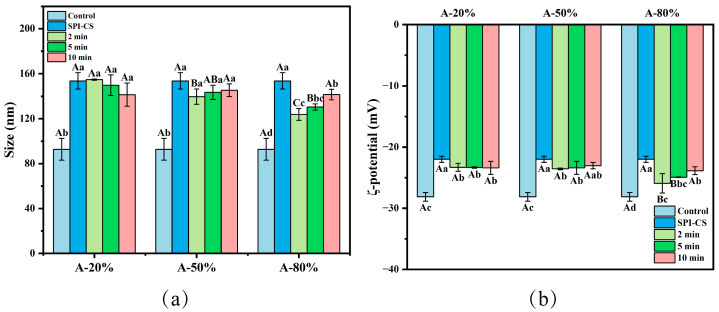
Effect of different ultrasound amplitude and ultrasound time on the particle size (**a**) and ζ-potential (**b**) of SPI–CS. A-20%, A-50%, A-80%: SPI–CS treated by 20%/50%/80% ultrasound amplitude at different duration times (2 min, 5 min, 10 min). Values with different letters indicate significant differences (*p* < 0.05), with uppercase letters representing differences between groups and lowercase letters representing differences within groups. Each legend clearly states that the data represent mean ± SE (*n* = 3).

Figure 1a clearly illustrated that the particle (coacervate) size associated with the SPI–CS system not exposed to ultrasonication was significantly higher by comparison with that measured for the control (raw SPI) (*p* < 0.05). Additionally, 80% ultrasound amplitude treatment significantly decreased the particle size of SPI–CS (*p* < 0.05). Ultrasound at 50% and 80% amplitude for 2 and 5 min significantly reduced the SPI–CS particle size (*p* < 0.05). No significant differences were detected among the 10 min samples at any amplitude. These results indicated that lower amplitude ultrasound was more favorable for decreasing the particle size of coacervates. Similarly, a marked reduction in particle size was also reported for zein–gum arabic coacervates following ultrasonic treatment [13]. This reduction was attributed to high hydrodynamic shear and forces cavitation effects induced by ultrasound, which most likely caused breaking the intramolecular and intermolecular noncovalent bonds, and thus reducing the particle size [14]. Furthermore, ultrasound might reduce the repulsion force and steric hindrance between molecules, also decreasing the size of the complexes [13]. At the same time, the particle size of A-50% and A-80% showed an increasing tendency with ultrasonication time, which was in line with the results reported by Wang et al. [15]. The results might be ascribed to the fact that the broken complexes further aggregate and rearrange to form larger assemblages [16].

The ζ-potential variation could reflect the strength of the electrostatic interaction of the protein-polysaccharide complex coacervate. By comparison with the control, all the other samples resulted after SPI coacervation with CS revealed absolute ζ-potential values significantly diminished (*p* < 0.05). These findings suggested that the negatively charged SPI was partially neutralized by the primary ammonium groups (positively charged) of CS via electrostatic interactions. Additionally, the ζ-potential absolute values of SPI–CS particles increased after ultrasound treatment (*p* < 0.05). This behavior was in line with the results reported elsewhere for the sodium caseinate–pectin complex, ultrasound-treated [17]. This aspect could be due to increasing the number of negatively charged moieties carried by the amino acid residues, such as aspartate and/or glutamate, oriented towards the outside of the protein structures, most likely largely unfolded under the ultrasound action. Another reasonable explanation could be related to the fact that the smaller coacervates might lead to larger surface areas, which could expose more and more surface charges [18]. However, when a longer sonication duration was applied, a decreased ζ-potential absolute value of A-80% was noticed. As in the case of the study reported by Wang et al. [19], this particular behavior could be explained by a complex process of particle reaggregation, with a direct consequence of reducing the absolute value of the ζ-potential.

### 3.2. Surface Hydrophobicity (H_0_) of SPI-CS Under Ultrasonic Treatment

**Figure 2:**
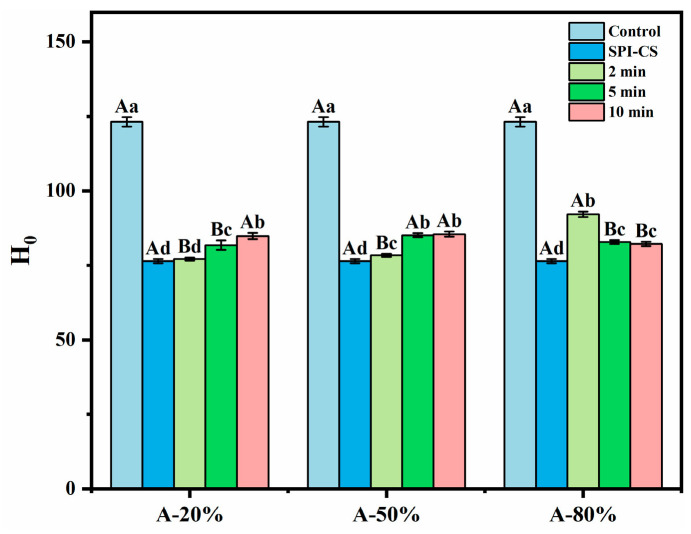
Effect of different ultrasound amplitudes and ultrasound times on the surface hydrophobicity of SPI–CS. Values with different letters indicate statistically significant differences (*p* < 0.05), with uppercase letters representing differences between groups and lowercase letters representing differences within groups.

The index of H_0_ is often used to assess the content of the hydrophobic groups exposed on the outside of a protein as a very important structural peculiarity of it. As illustrated in Figure 2, the presence of CS in tandem with SPI in the studied systems caused a significant decrease (*p* < 0.05) in their H_0_ values by comparison with H_0_ of the control (*p* < 0.05) due to the fact that the hydrophobic sites on the protein (SPI) surface were covered by the polysaccharide (CS) [20]. In addition, the ultrasound treatment significantly improved the H_0_ of SPI–CS (*p* < 0.05). Similar results concerning H_0_ for soy protein isolate–citrus pectin electrostatic complexes under ultrasound treatment [21] and H_0_ for whey protein isolate–chitosan/chitooligosaccharide complex under the treatment of ultrasound [22] were also reported. This phenomenon was attributed to the physico-mechanical effects generated during ultrasound treatment, whereby a mass transfer process was induced, and the hydrophobic moieties initially buried in the non-denatured protein were transferred to the protein surface as a result of unfolding [22]. In addition, the H_0_ values for A-20% and A-50% exhibited an increasing ultrasound time-dependence, while the H_0_ for A-80% showed an opposite trend. These findings were in accordance with what Wang et al. [15] reported for the rice bran protein–chlorogenic acid complex: H_0_ follows an increasing ultrasonication time course under lower ultrasound amplitude and an opposite trend under higher ultrasound amplitude. Practically, such an in-excess ultrasonication could cause particles to associate via hydrophobic regions exposed to the aqueous environment, leading to aggregates superficially less hydrophobic (or even hydrophilic) as suggested by Zhao et al. [23].

### 3.3. Fluorescence Spectroscopy of SPI-CS Under Ultrasonic Treatment

**Figure 3:**
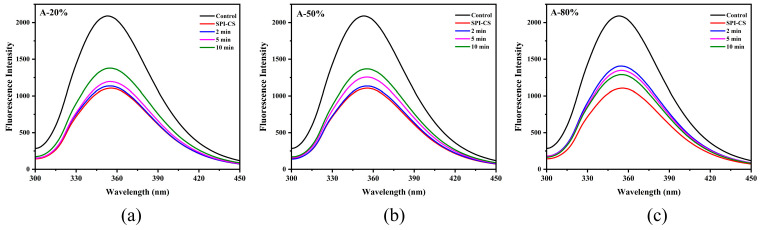
Effect of different ultrasound amplitudes and ultrasound times on the fluorescence spectra of SPI–CS (A-20 % (**a**), A-50 % (**b**), A-80 % (**c**)).

Intrinsic tryptophan fluorescence is highly sensitive to the polarity and dynamics of the local microenvironment of tryptophan residues in proteins and is therefore widely used to monitor changes in protein tertiary structure. Upon combining with CS, the fluorescence intensity of SPI markedly decreased (Figure 3), suggesting that tryptophan residues were transferred from a more polar to a more hydrophobic environment [24]. Ultrasound treatment subsequently increased the fluorescence intensity of SPI–CS, indicating partial alteration of the tryptophan environment under sonication. Similar results of fluorescence spectrum appeared in the previous work for sodium caseinate–pectin complex under ultrasound treatment [17] and whey protein isolate–chitosan/chitooligosaccharide complex under the ultrasound treatment [22]. Additionally, the fluorescence spectra intensity of A-20% and A-50% showed an upward trend, while the fluorescence spectra intensity of A-80% showed a downward trend, which was consistent with the aforementioned description regarding surface hydrophobicity.

### 3.4. FTIR Spectroscopy and Protein Secondary Structure

**Figure 4:**
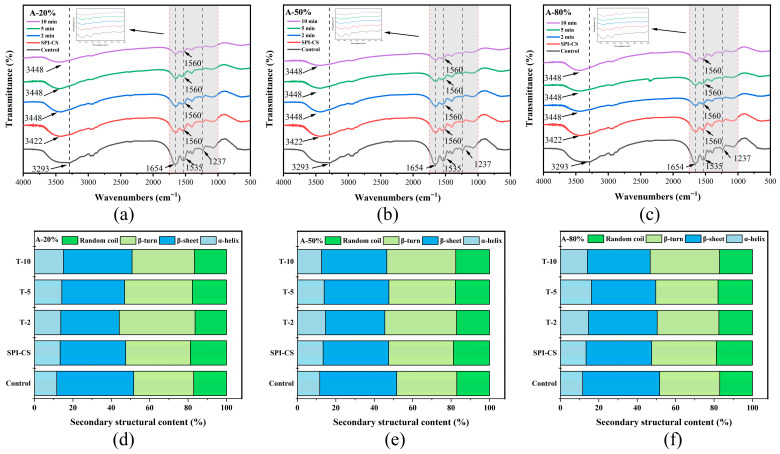
FTIR spectra (**a**–**c**) of SPI and SPI–CS complexes and content of different types of secondary structures (**d**–**f**) of the protein component for the studied systems under specified working parameters.

FTIR spectroscopy is a powerful tool useful to explore interactions between proteins and polysaccharides. In the FTIR spectra, the bands in the 1600–1700 cm^−1^ region are assigned to the amide I vibration of SPI, mainly arising from C=O stretching of peptide linkages, whereas the band at around 1535–1560 cm^−1^ is attributed to the amide II vibration (N–H bending and C–N stretching) together with the bending of protonated amino groups (–NH_3_^+^) in chitosan (Figure 4a–c). The shift and intensity changes in these bands after complexation and ultrasound treatment indicate modified electrostatic interactions between SPI and CS, in agreement with previous reports on protein–chitosan complexes [2,25]. In addition, the broad band between 3290 and 3450 cm^−1^ corresponds to overlapped O–H and N–H stretching vibrations, reflecting extensive intra- and intermolecular hydrogen bonding, and its evolution upon complexation and ultrasound treatment suggests a rearrangement of the hydrogen-bonding network in the SPI–CS coacervates [26]. A similar result was found in the FTIR spectrum of zein–chitosan complex coacervates under the treatment of ultrasound [10]. Moreover, SPI–CS samples treated at different ultrasound amplitudes and times exhibited similar overall band patterns with only changes in peak intensity, implying that non-covalent interactions remained the dominant forces governing complex formation [22].

Secondary-structure compositions were estimated from FTIR spectra by deconvolution of the amide I region (1600–1700 cm^−1^), with bands at 1610–1640 and 1680–1690 cm^−1^ assigned to β-sheet, 1640–1650 cm^−1^ to random coil, 1650–1660 cm^−1^ to α-helix, and 1660–1680 cm^−1^ to β-turn. Notably, the β-sheet content of the SPI–CS remarkably declined, with a corresponding shift toward α-helix and β-turn (Figure 4d–f). Moreover, no discernible differences were observed in the random coil’s content. The possible reason was that the intermolecular interactions of the native SPI were impaired, resulting in the deduction of the β-sheet content. The result may be associated with the enhancement of hydrogen bonding after ultrasonic treatment [27]. Moreover, the SPI–CS β-turn content was increased after different ultrasound amplitudes and times, which might be because the ultrasound makes the complex structure looser [28]. The α-helix content increased markedly, whereas the β-sheet content showed a corresponding decrease. This was homologous with the results of secondary structure directed by Tang et al. [29] and Yang et al. [30]. Additionally, the strengthening of electrostatic and hydrogen-bonding interactions in SPI–CS coacervates was reflected by an increased amide I band intensity and a structural transition from β-sheet to α-helix and β-turn conformations, which was consistent with observations for β-lactoglobulin–basil seed gum mixtures [31]. The results showed that ultrasound treatment enhanced the interaction between SPI and CS, which was in line with the above FTIR results.

### 3.5. F-SH

Table 1 showed a notable reduction in the F-SH content of SPI–CS (ultrasound non-treated) compared to the control system (*p* < 0.05). This result might suggest some structural changes undergone by SPI after its coacervation with CS, when CS could partially shield the exposed F-SH groups of the protein component. In addition, the F-SH content of SPI–CS under 20%, 50%, and 80% ultrasound amplitude at 2 min markedly increased (*p* < 0.05). Such an aspect is in line with what Zhao et al. [32] previously reported. This was mainly due to the cavitation effect induced by ultrasound that breaks disulfide bonds, generating new SH groups [32], on one hand, and unfolds protein conformations, leading to exposing SH groups from the inside to the outside of the protein [18,32,33], on the other hand. Furthermore, the F-SH content of A-50% and A-80% showed a decreasing trend, which was in agreement with the results reported elsewhere on gliadin aggregates in wheat and green wheat [33]. A behavior as described above could be due to the fact that the free sulfhydryl groups at higher ultrasound amplitudes and longer ultrasound times were chemically involved in intramolecular and intermolecular disulfide bonds [34]. The formation occurred as a result of the possible oxidation reactions initiated by oxidizing species generated in the aqueous phase during ultrasonication [18,34,35].

### 3.6. Solubility

Solubility is not only an effective indicator of protein conformational changes and aggregation characteristics, but it is also closely related to the functional characteristics of the protein. The marked reduction in SPI solubility after coacervation with CS was attributed to the larger particle size and decreased net charge of the complexes, which were considered to promote aggregation and limit dispersion (Table 2). Similar solubility reductions were reported for other protein–polysaccharide coacervates, such as sesame protein isolate–tragacanth gum systems [36]. In addition, 20% and 50% ultrasound amplitude at 10 min, and 80% ultrasound at 2 min and 5 min markedly increased the solubility of the SPI–CS when compared to the non-ultrasonicated complexes (*p* < 0.05). Similar results were reported on the solubility of myofibrillar protein–konjac glucomannan complex under ultrasound treatment [37]. A possible explanation for that could be a disintegration process of the aggregates into smaller ones, the process induced by the ultrasound action, with a very favorable effect on increasing the solubility of the newly formed complexes [38,39]. In addition, the solubility of A-20% and A-50% showed an increasing ultrasound time dependence, while the solubility of A-80% exhibited a decreasing trend. This reflects a complex behavior that might be related to dynamic changes involving intermolecular hydrophobic interactions and disulfide bond formation, with consequences in particle size evolution and, in turn, in aggregate solubility [32].

### 3.7. Emulsifying Properties of SPI-CS Under Ultrasonic Treatment

**Figure 5:**
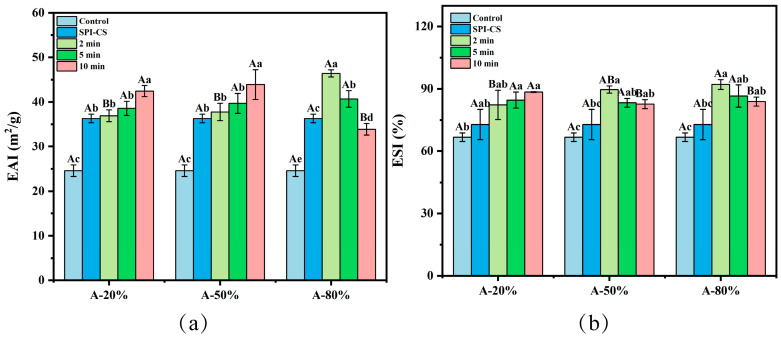
Effect of different ultrasound amplitude and ultrasound time on the EAI (**a**) and ESI (**b**) of SPI–CS. Values with different letters indicate significant differences (*p* < 0.05), with uppercase letters representing differences between groups and lowercase letters representing differences within groups.

Emulsifying property, as a crucial indicator of plant-based proteins, has attracted extensive attention in protein functional applications. As depicted in Figure 5a, CS significantly increased the emulsifying activity index (EAI) of the SPI–CS (ultrasound non-treated) by comparison with that of the control, which means that both SPI and CS possess a high emulsifying ability, displaying a synergistic effect regarding the emulsifying property when it comes to SPI–CS complexes [40]. Previous studies shown that protein–polysaccharide coacervate complexes can exhibit enhanced adsorption at the droplet interface, improved interfacial film viscoelasticity, and increased steric stabilization of emulsions [20,41]. These reports provide a mechanistic context for the higher EAI observed for the SPI–CS complexes in the present study. In addition, ultrasound treatment further dramatically increased the EAI of SPI–CS (*p* < 0.05), except for A-80% at 10 min of ultrasonication. These findings are similar to the results reported by Shen et al. in studying whey protein isolate [42]. This effect may be attributed to ultrasound-induced increases in the surface hydrophobicity of the complexes, which strengthen hydrophobic interactions and promote their adsorption at the oil–water interface, thereby improving their emulsifying activity [43,44]. Moreover, the particle size reduction in the complex by ultrasound was favorable for improving the adsorption capacity of the complex at liquid–liquid interfaces, which is equivalent to enhanced emulsifying properties [42]. Aforementioned results of surface hydrophobicity and particle size also supported this view. Additionally, the A-20% and A-50% showed an upward evolution with ultrasonication time, while A-80% displayed an opposite trend. Similarly, the EAI of konjac glucomannan–peanut protein under the ultrasound treatment showed a downward dependence on ultrasonication time [45]. This might be due to breaking complexes into smaller pieces followed by their rearrangement into larger aggregates with a lower emulsifying capacity and, eventually, with decreasing the EAI values [17].

The emulsifying stability index (ESI) of SPI was notably increased after coacervation (*p* < 0.05) (Figure 5b). The increase in the emulsifying stability index (ESI) after coacervation was likely related to the formation of a thicker protein–polysaccharide layer around the oil droplets, which provides steric hindrance and slows droplet aggregation in the dispersed phase [42]. Ultrasound treatment further promoted interfacial adsorption of the SPI–CS complexes and the build-up of such protective layers, thereby contributing to the improved emulsion stability [46]. In addition, the ESI value for SPI–CS under the treatment of ultrasound prominently rose (*p* < 0.05), which was in line with the results reported elsewhere for ESI of the whey protein isolate–chitosan/chitooligosaccharide complex [22]. A plausible reason was that ultrasound promoted the complex adsorption at the oil/water interface, and steric hindrance formation around the emulsion droplets [47]. In other words, this was equivalent to saying that the ultrasound treatment, which could inhibit the aggregation of droplets and delay phase separation, resulted in improved emulsion stability [3]. On the other hand, the upward evolution of ESI with the sonication time for A-20% system and the opposite trend noticed at A-50% and A-80% might be explained by the fact that high amplitude ultrasound could affect the complexes’ distribution at the oil–water interface, leading to conformational rearrangements associated with decreasing ESI values [15].

### 3.8. Flavor Profile

**Figure 6:**
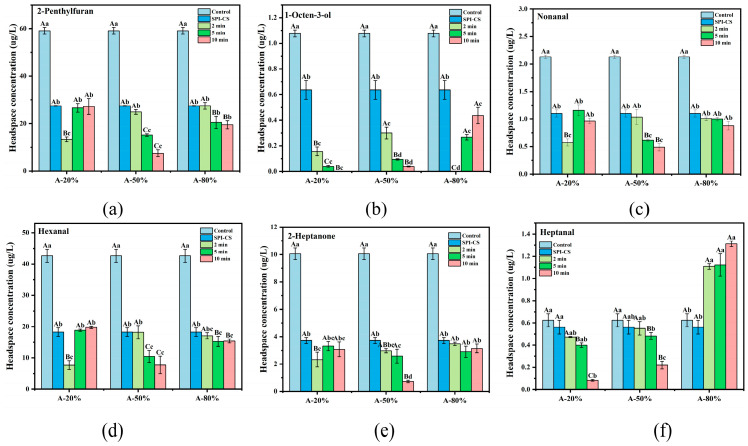
Identification and quantification of representative beany-flavor compounds in SPI–CS using HS-SPME-GC-MS. 2-Penthylfuran (**a**), 1-octen-3-ol (**b**), nonanal (**c**), hexanal (**d**), 2-heptanone (**e**), and heptanal (**f**). Values with different letters indicate significant differences (*p* < 0.05), with uppercase letters representing differences between groups and lowercase letters representing differences within groups.

Plant-based proteins were restricted by beany flavor due to the negative impact on sensory and consumer science. Six representative beany flavor compounds—2-penthylfuran (beany off-flavors), 1-octen-3-ol (mushroom), nonanal (green, fatty), 2-heptanone (fragrance), hexanal (cut grass), and heptanal (dry fish)—were selected for quantitative analysis. As shown in Figure 6, the beany-flavor release concentrations of the SPI were dramatically decreased after coacervation with CS (*p* < 0.05). Additionally, it is also likely that the ultrasound treatment significantly further diminished the beany-flavor release of SPI–CS (*p* < 0.05). Ji et al. [48] also found similar results. In general, interactions between SPI and aldehydic beany-flavor compounds were mediated by both hydrophobic interactions and hydrogen bonding, whereas ketone and furan compounds were also involved in hydrophobic interactions and acted as hydrogen-bond acceptors toward suitable donor groups on the protein [49]. Thus, the reduced release of beany-flavor compounds after ultrasound treatment may be attributed to the greater exposure of hydrophobic regions on the SPI–CS complexes, which strengthens hydrophobic contacts and hydrogen bonding with aldehyde, ketone, and furan compounds, in agreement with the increased H0 observed for ultrasonicated SPI–CS. The above results of surface hydrophobicity also support this view. Furthermore, the SPI–CS coacervates fragmented and unfolded under the ultrasound provide more and more hydrophobic and hydrogen bonding sites for binding the beany flavor compounds [50]. Another possible contribution to the reduced beany off-flavor was that carbonyl-containing compounds, such as aldehydes (hexanal and nonanal) and heptanone, formed covalent adducts with free sulfhydryl groups in the SPI–CS complexes, thereby lowering the level of free beany-flavor compounds [35].

In addition, the release concentration of 1-octen-3-ol and heptanal of SPI–CS under the 80% ultrasound amplitude showed an increasing trend with sonication duration. These results suggested that partial reaggregation of SPI–CS coacervates at high ultrasound intensity might have buried active binding sites, thereby reducing the overall adsorption capacity of the complexes for these flavor compounds [51]. Notably, the samples treated with ultrasound at 20% and 50% amplitude for 10 min exhibited the best effect of mitigation of beany flavor compounds among all the SPI–CS samples. This seems to suggest that longer ultrasound time at lower ultrasound amplitude is more conducive to mitigating the beany flavor. The 2-penthylfuran, nonanal, 2-heptanone, and hexanal showed the lowest release concentration at the 50% amplitude under 10 min ultrasound; while the 1-octen-3-ol and heptanal showed the lowest release concentration at the 20% amplitude under 2 min. Additionally, ultrasonic treatment reduced the release concentration of beany flavor in SPI–CS by more than 50%.

## 4. Conclusions

In this work, the impact of ultrasound on the structure, functionality, and flavor behavior of SPI–CS coacervates was systematically evaluated. Coacervation with CS was found to increase particle size, decrease solubility, and enhance emulsifying activity and stability, while markedly reducing the release of representative beany-flavor compounds compared with SPI alone. Ultrasound treatment further decreased particle size, increased the absolute ζ-potential and surface hydrophobicity, and induced a redistribution of secondary structures from β-sheet toward α-helix and β-turn, indicating that electrostatic, hydrogen-bonding, hydrophobic, and disulfide-related interactions within SPI–CS coacervates were modulated. These structural changes were accompanied by generally improved solubility and emulsifying indices, particularly at moderate amplitudes (20%, 50%) and longer sonication times, which were most effective in enhancing emulsifying activity and suppressing beany flavor release, whereas prolonged treatment at 80% amplitude resulted in partial reaggregation and compound-dependent flavor behavior. Although ultrasound-induced reductions in particle size were associated with improved emulsifying properties, the molecular-weight distribution of the complexes was not quantified, so a direct link between changes in molecular weight and emulsifying behavior could not be established. In future work, this relationship should be clarified by characterizing the molecular-weight distribution of the complexes using techniques such as gel permeation chromatography (GPC) and SDS-PAGE. Interfacial adsorption and interfacial film viscoelasticity were inferred rather than directly measured, so subsequent studies should include interfacial tension and interfacial rheology to clarify the interfacial role of SPI–CS complexes, as well as address scale-up issues related to energy input and temperature control under industrial ultrasound conditions.

## Figures and Tables

**Table 1:** Effect of different ultrasound amplitudes and ultrasound time on the content of F-SH of SPI–CS.

F-SH Content (μmol/g)			
	A-20%	A-50%	A-80%
Control	7.21 ± 0.38 ^Aa^	7.21 ± 0.38 ^Aa^	7.21 ± 0.38 ^Aa^
SPI–CS	5.69 ± 0.43 ^Ab^	5.69 ± 0.43 ^Abc^	5.69 ± 0.43 ^Abc^
2 min	6.03 ± 0.41 ^Ab^	5.99 ± 0.26 ^Ab^	6.23 ± 0.22 ^Ab^
5 min	5.52 ± 0.12 ^Bb^	5.62 ± 0.07 ^Bbc^	5.83 ± 0.08 ^Abc^
10 min	5.50 ± 0.47 ^Ab^	5.37 ± 0.30 ^Ac^	5.54 ± 0.21 ^Ac^

Values with different letters indicate significant differences (*p* < 0.05), with uppercase letters representing differences between groups and lowercase letters representing differences within groups.

**Table 2:** Effect of different ultrasound amplitudes and ultrasound times on the solubility of SPI–CS.

Solubility (%)			
	A-20%	A-50%	A-80%
Control	46.29 ± 1.28 ^Aa^	46.29 ± 1.28 ^Aa^	46.29 ± 1.28 ^Aa^
SPI–CS	38.96 ± 0.84 ^Ab^	38.96 ± 0.84 ^Ac^	38.96 ± 0.84 ^Ac^
2 min	39.82 ± 2.08 ^Ab^	39.60 ± 3.75 ^Ac^	43.81 ± 0.19 ^Aab^
5 min	40.20 ± 1.96 ^Ab^	41.15 ± 1.43 ^Abc^	40.51 ± 4.17 ^Abc^
10 min	45.77 ± 4.08 ^Aa^	44.74 ± 3.38 ^Aab^	33.36 ± 1.83 ^Bd^

Values with different letters indicate significant differences (*p* < 0.05), with uppercase letters representing differences between groups and lowercase letters representing differences within groups.

## Data Availability

The original contributions presented in the study are included in the article; further inquiries can be directed to the corresponding authors.
